# Genomics and conservation units: The genetic basis of adult migration timing in Pacific salmonids

**DOI:** 10.1111/eva.12687

**Published:** 2018-08-28

**Authors:** Robin S. Waples, Steven T. Lindley

**Affiliations:** ^1^ NOAA Fisheries Northwest Fisheries Science Center Seattle Washington; ^2^ NOAA Fisheries Southwest Fisheries Science Center Santa Cruz California

**Keywords:** adaptation, conservation genetics, fisheries management, genomics, life history evolution, natural selection, population genetics

## Abstract

It is now routinely possible to generate genomics‐scale datasets for nonmodel species; however, many questions remain about how best to use these data for conservation and management. Some recent genomics studies of anadromous Pacific salmonids have reported a strong association between alleles at one or a very few genes and a key life history trait (adult migration timing) that has played an important role in defining conservation units. Publication of these results has already spurred a legal challenge to the existing framework for managing these species, which was developed under the paradigm that most phenotypic traits are controlled by many genes of small effect, and that parallel evolution of life history traits is common. But what if a key life history trait can only be expressed if a specific allele is present? Does the current framework need to be modified to account for the new genomics results, as some now propose? Although this real‐world example focuses on Pacific salmonids, the issues regarding how genomics can inform us about the genetic basis of phenotypic traits, and what that means for applied conservation, are much more general. In this perspective, we consider these issues and outline a general process that can be used to help generate the types of additional information that would be needed to make informed decisions about the adequacy of existing conservation and management frameworks.

## 1 INTRODUCTION

**Figure 1:**
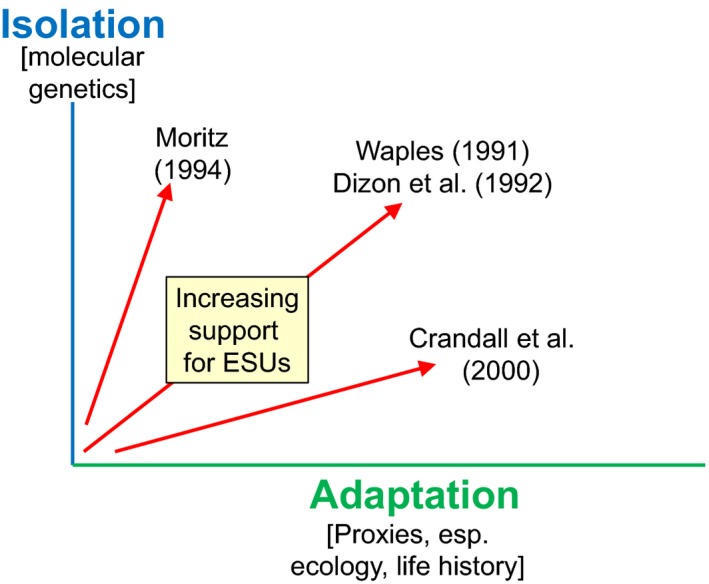
A general framework for evaluating strength of evidence in support of ESUs or other types of conservation units. Widely used ESU concepts focus on two axes of intraspecific diversity (isolation and adaptation) but differ in the relative importance assigned to each. Moritz's (1994) reciprocal monophyly of mtDNA concept focused almost entirely on isolation; the exchangeability concept proposed by Crandall, Bininda‐Emonds, Mace, and Wayne (2000) placed more emphasis on adaptation; and the frameworks developed by Waples (1991) and Dizon, Lockyer, Perrin, Demaster, and Sisson (1992) placed roughly equal weight on each factor. Until recently, information regarding isolation generally relied on molecular genetic data, whereas inferences about adaptations typically had to be based on proxies such as ecology, behavior, life history, and other phenotypic traits. Recent advances in genomics technology for non‐model species now make it possible to identify genes associated with traits thought to be adaptive—but is this sufficient to adequately characterize this axis?

Advances in genomics technologies have revolutionized many areas of biology, and are beginning to be taken up in conservation (Benestan et al., 2015; Bernatchez, 2016; Garner et al., 2016; Hendricks et al., 2018; Moore, Bourret et al., 2014; Shafer et al., 2015; Waters et al., 2018). Identification of intraspecific conservation units (CUs) is one of the most common applications of genetic data. Two levels of CUs are typically identified (Moritz, 1994): Evolutionarily Significant Units (ESUs), which represent important evolutionary/ecological components of the species as a whole, and Management Units (MUs), which typically represent demographically independent units that merit separate management. Several frameworks for defining ESUs have been proposed (reviewed by Fraser & Bernatchez, 2001), but most can be characterized in terms of the relative importance they place on two major axes of diversity: isolation and adaptation (Waples, 2006; Figure 1). Prior to the last decade, genetic data for markers thought to be selectively neutral were used primarily to characterize the isolation axis, whereas insights regarding adaptations had to rely primarily on proxies, such as ecological features of the species' habitats, or phenotypic traits that reflect combined effects of genetics and the environment.

Genomics data potentially can help to quantify both types of diversity (Funk, McKay, Hohenlohe, & Allendorf, 2012). The ability to assay tens or hundreds of thousands of DNA markers greatly enhances power to infer historical demography and patterns of connectivity, which in turn increases resolution along the isolation axis (e.g., Benestan et al., 2015). Genomics methods also can identify genes associated with phenotypes thought to be adaptive. As demonstrated by Moore, Bourret et al. (2014), there is no guarantee that this new information will suggest any major changes to conservation units defined on the basis of neutral markers. On the other hand, it is inevitable that sometimes different patterns of genetic affinity will be implied by neutral and putatively adaptive markers. Given that the goal of identifying and protecting CUs is the conservation of intraspecific biodiversity, in at least some of these cases it might be necessary to consider whether to revise existing conservation units based on the genomics data, and if so how best to do so.

A recent example of this latter issue involves conservation units of Pacific salmon (*Oncorhynchus* spp.) and steelhead (the anadromous form of rainbow trout, *O. mykiss*). Roughly one‐third of the Pacific salmon and steelhead populations that existed in the coterminous United States ca 1800 have been extirpated (Gustafson et al., 2007), and about half of those that remain are federally listed as threatened or endangered under the U.S. Endangered Species Act (ESA) (http://www.westcoast.fisheries.noaa.gov/protected_species/salmon_steelhead/salmon_and_steelhead_listings/salmon_and_steelhead_listings.html). These populations are protected under a provision of the ESA that allows listing of Distinct Population Segments (DPSs) of vertebrate species. Pacific salmon populations or groups of populations are considered DPSs if they meet the criteria to be an ESU (NMFS 1991; Waples, 1991). In Canada, formal assessments under the Species at Risk Act (SARA) are not complete, but a number of populations or groups of populations of Pacific salmon and steelhead have been federally listed as Designatable Units (DUs), which are roughly equivalent to DPSs. Salmon are also at risk on the Atlantic coast of North America, where only 4 of 15 recognized Canadian DUs of Atlantic salmon (*Salmo salar*) are considered “not at risk” under SARA (COSEWIC 2010). In the United States, all historic Atlantic salmon populations have been extirpated except those in Maine, which are listed as Endangered under the ESA (USFWS and NMFS 2000).

In the United States, the framework used to identify listable units for Pacific salmon places roughly equal weight on reproductive isolation and adaptation (Waples, 1991, 2006; Figure 1). Atlantic salmon and steelhead are dealt with under a broader DPS framework that applies to all vertebrate species (USFWS and NMFS, 1996), but its two DPS criteria (discreteness and significance) are largely parallel to those in the salmon policy. After initially experimenting with a different approach for defining DUs under SARA (Green, 2005), in 2009 Canada adopted a framework that is essentially identical to the discreteness/significance approach used to define DPSs in the United States (COSEWIC 2018).

Proxies most commonly used for adaptive differences/significance have been phenotypic and life history traits and distinctive ecological features of the habitat (Waples, 2006). Of the former, adult migration timing (season of entry into fresh water to begin the spawning migration) has received particularly careful scrutiny, not only because of clear evidence for a genetic basis (Carlson & Seamons, 2008), but also because this trait has been widely used to define harvest management and artificial propagation programs in both the United States and Canada. The strongest adult migration contrast is between early returning populations (summer steelhead or spring Chinook salmon, *O. tshawytscha*), which enter fresh water when they are immature and hold for many months before spawning, and late‐returning populations (winter steelhead or fall Chinook salmon), which mature in the ocean and spawn soon after entering fresh water. Maintaining diversity among populations in migration timing and other life history traits has been shown to be important for long‐term persistence and sustainability (Moore, Yeakel, Peard, Lough, & Beere, 2014; Schindler et al., 2010).

**Figure 2:**
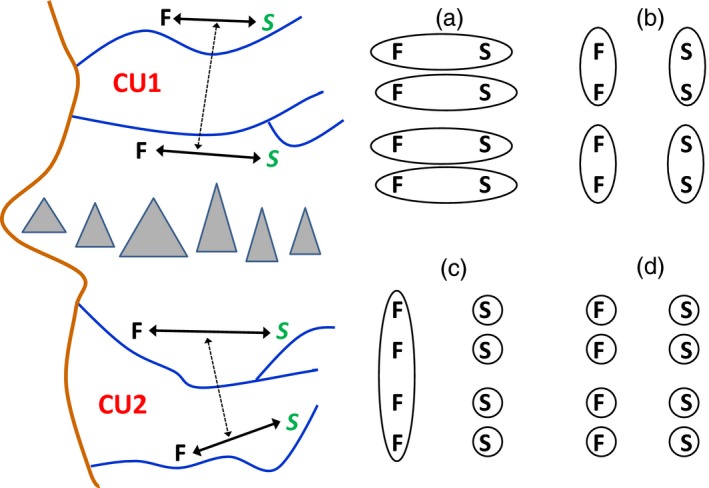
Left panel: Schematic diagram of geographic and evolutionary relationships among aquatic populations with different life history traits. Each of four rivers (blue lines) supports two life history types (*S*, F). The thick brown line is the coastline, and gray triangles indicate an ecological break that also serves as a partial isolating mechanism. The ecological differences, together with overall genetic affinities within and among rivers (black arrows), lead to division of the area into two conservation units (CU1, CU2). However, at one small region of the genome, all of the *S* populations share a single “green” allele. Does this require a change in how the conservation units are defined? If so, what should the new configuration look like? Right panel: Four (of many) alternative CU scenarios. (a) Each river is a separate CU that contains both life history types; (b) Life history types define separate CUs; (c, d) Each *S* population is in a separate CU, with the F populations either all lumped together (c) or also in separate CUs (d)

A considerable body of evidence has suggested that differences in adult migration timing often have resulted from parallel adaptations and hence do not define separate evolutionary lineages. Studies using presumably neutral genetic markers have shown that, in most coastal drainages from California to Washington, Chinook salmon and steelhead populations from the same river that have different run timing are genetically more similar to each other than are populations from different rivers that have the same run timing (Figure 2). This pattern was first documented with allozymes (Chilcote, Crawford, & Leider, 1980; Waples, Teel, Myers, & Marshall, 2004) and has subsequently been confirmed in microsatellites and single‐nucleotide polymorphisms (SNPs) (Arciniega et al., 2016). These results have led to the conclusion that run‐timing diversity has arisen many times within each species by a process of parallel evolution. It is generally assumed that the late‐maturing life history is the more general form, and the early returning form evolves from standing genetic variation only when suitable habitat/environmental conditions are present (e.g., seasonal access to high‐elevation habitats and suitable locations for summer holding). This view is consistent with the reigning paradigm in quantitative genetics, which has been that most phenotypic traits are controlled by many genes of small effect. A classic example of this is the genetic architecture of height in humans. Studies of relatives consistently indicate that about 80% of variation in human height is due to additive genetic factors, yet the top ~10,000 SNPs most strongly associated with human height still explain only about 30% of the phenotypic variance (Wood et al., 2014).

From the perspective of the polygenic, quantitative genetics paradigm, this means that each time the early run phenotype evolves from the late‐run phenotype, this life history “problem” could potentially be solved in a new way by mobilizing different sets of the many genes that can modulate migration timing (see Bernatchez, 2016 for similar examples in other species). Most ESUs of Pacific salmon and steelhead reflect this paradigm: if nearest relatives are determined more by geography than phenotype, a conservation unit that, for example, included all spring (but not fall) Chinook salmon populations would be artificial and not reflect evolutionary lineages (Figure 2). With only a few exceptions, therefore, differences in adult run timing have been considered important components of diversity within ESUs of Pacific salmon and steelhead, rather than features that defined separate ESUs.

Some recent genomics studies provide a novel perspective on the evolution of adult migration timing. Hess, Zendt, Matala, and Narum (2016) used genome‐wide association mapping of over 15,000 SNP markers to study the adult migration phenotype in a Columbia River steelhead population that exhibits bimodal migration timing. Summer steelhead return from May to October and winter steelhead arrive from December to May; both forms spawn in late winter or spring. In spite of the extensive interbreeding that limits neutral genetic differences between the two forms, Hess et al. (2016) identified three SNPs from a GREB1‐like gene that explained 46% of the variation in adult migration timing in the population they studied. More recently, Prince et al. (2017) surveyed several hundred thousand SNPs in a number of coastal populations of both steelhead and Chinook salmon from Washington, Oregon, and California; their results simultaneously confirmed and called into question the reigning parallel evolution paradigm. At ~99.99% of the markers examined, Prince et al. (2017) found evolutionary relationships typical of the existing paradigm: genetic affinities reflected geography rather than adult migration timing, and populations formed genetic clusters that reflected current ESU structure in both species. On the other hand, in the vicinity of a small region of the GREB1‐like gene in each species, Prince et al. reported that specific alleles are strongly associated with populations characterized by early (premature) migration timing (summer steelhead or spring Chinook salmon). Moreover, Prince et al. (2017) argued that the key mutations arose only once within each species and spread by migration and positive selection. Finally, the authors concluded that the existing ESU framework for these species (which assumes that parallel evolution of life history traits is relatively common) might not be adequate to conserve important components of diversity. If early migration timing can only evolve when specific alleles are present, a supplemental framework to ensure conservation of those genes might be necessary.

Disentangling the genetic basis of adult migration timing in salmon is not merely an academic problem. The early returning, premature‐migrating forms of Chinook salmon and steelhead have experienced disproportionately high rates of local extirpation (Gustafson et al., 2007; Quinn, McGinnity, & Reed, 2016), largely because their specialized life history and habitat requirements make them particularly vulnerable to some anthropogenic sources of mortality, and especially to effects of dams that block access to holding and spawning habitats. Soon after publication of the Prince et al. (2017) report, the U.S. federal government received a formal ESA petition to list spring Chinook salmon from the upper Klamath and Trinity rivers in California as a separate ESU (NMFS 2018). Currently, those populations are considered part of a larger ESU that also includes many fall‐migrating populations in the Klamath River basin, and because of the latter's relatively high overall abundance, the ESU is not federally protected.

The new genomics results raise some important questions about conservation and management priorities—in particular, the relative importance in conservation of focusing on (a) preserving certain phenotypes, or (b) conserving key ecological/evolutionary processes that are ultimately responsible for creating diversity (Moritz, 2002). If evolution of different life histories is a common parallel process, it is particularly important to conserve the ability of natural ecological and evolutionary processes to produce variation capable of sustaining species into the future. But if expression of certain life history traits is only possible if individuals carry a specific gene or genes, it might be necessary to include at least some aspects of a more typological approach, to ensure that genetic variants capable of producing the phenotypes are maintained.

Do these recent genomics results indicate that any changes are needed to current conservation/management practices for Pacific salmon and steelhead? That is a complex question with no simple answers, at least at present. Below we identify a series of questions that should be addressed to provide a more informed basis for making management decisions.

## 2 KEY QUESTIONS

1. What is the distribution of genetic variation for adult migration timing in space and time?
2. Are the genes identified by Hess et al. (2016) and Prince et al. (2017) actually responsible for adult migration timing, and if so by what mechanism?
3. What is the pattern of dominance at the GREB1‐like gene? What phenotype do heterozygotes express, and what is their fitness compared to homozygotes?
4. Prince et al. (2017) argued that the gene(s) associated with early migration timing evolved only once within each species. Is that the case, or are the genetic variants more evolutionarily labile?
5. Do the genes associated with migration timing have the same effect in populations inhabiting different environments and with different genetic backgrounds?
6. The empirical data show that the vast majority of the markers surveyed show a pattern of genetic affinity based on geography, whereas one small area, reflecting perhaps a single gene, shows a strong association between genotype and migratory phenotype. What series of events/processes could produce a result like that, and what sort of testable hypotheses would be generated (e.g., signatures of selective sweeps/bottlenecks) if that is how things happened?
7. How common is this phenomenon? Is it likely that strong associations will be found between specific alleles and many other phenotypic/life history traits?
8. Finally, what procedures are already in place to help protect diversity (genetic, phenotypic, ecological) within ESUs? Are these frameworks still robust in the context of the new results and answers to the above questions, or are changes needed?

## 3 DISCUSSION

Applied conservation typically requires one to balance trade‐offs between acting in the face of considerable uncertainty and delaying until more information is available. Although answers to each of the above questions could appreciably reduce uncertainty associated with making decisions about conservation units for Pacific salmon and steelhead, the questions are not all of equal importance, and addressing some will be much harder, and take much longer, than others.

Question 1 (distribution of genetic variation) is arguably the most important to tackle first. Hess et al. (2016) studied a single population of one species, and Prince et al. (2017) focused exclusively on populations known to express extreme forms of migration timing diversity. Now, it is crucial to obtain a more comprehensive picture of the genetic makeup of populations throughout each species' range, or at a minimum along the Pacific Coast of North America. In both species, populations can be found that enter fresh water during every month of the year. Even if some populations are largely fixed for one migration timing allele or another, are other populations more polymorphic, in which case they might serve as reservoirs for important genetic variants? Ideally, new analyses would include both phenotypic and genetic data for the same individuals; in the Prince et al. (2017) study, genetic data for individuals were compared to generic run‐timing designations for the populations of origin. Question 3 (expression in heterozygotes) is also important in this context. If the allele for early migration timing is recessive, it could exist at relatively high frequency in other populations because heterozygotes do not experience a fitness cost. Even if the heterozygote phenotype is intermediate, the fitness consequences could differ among populations, depending on local ecological/environmental conditions (cf Question 5).

Fortunately, Question 1 also should be easiest to gain substantial new insights into, using existing techniques and (in part at least) existing samples. A number of laboratories are already conducting genomics analyses of Chinook salmon and/or steelhead, and many have tissue or DNA samples that could be analyzed with this question in mind. Some new information relevant to Questions 1 and 3 has already been compiled (Narum, Di Genova, Micheletti, & Maass, 2018; Thompson et al., 2018). Conversely, although Question 2 is obviously of considerable importance (correlation after all does not establish cause and effect), addressing it is logistically challenging for natural populations with life histories like Pacific salmon, so it probably will be a number of years before definitive answers can be obtained.

The answer to Question 4 (mutational history) cannot be obtained by direct observation, so it has to be inferred from empirical patterns in the data. It will be important to see whether a consensus emerges in the published literature regarding the claim by Prince et al. (2017) that the mutant alleles arose only once in each species.

Question 6 (identifying a realistic scenario that could have produced the empirical data) is hypothetical but nevertheless crucial to tackle. If the new genomics data really do imply a new paradigm for understanding evolution of key life history traits, it is essential to provide biologists, managers, and policy makers with a clear understanding of how the proposed processes might have operated in the real world. That is, what is needed are one or more plausible scenarios that explain in plain language how a combination of evolutionary forces (mutation, migration, natural selection, genetic drift) could have produced the distribution of adult migration phenotypes we currently observe in Chinook salmon and steelhead.

Understanding how often genes of large effect are expected to occur (Question 7) is important to provide a broader perspective for evaluating results reported by the two genomics papers. Genomics methods have facilitated identification of genes of large effect in natural populations of a wide range of species (Nadeau & Jiggins, 2010). If salmon and steelhead conservation units are adjusted to account for specific genetic variants associated with adult migration timing, what happens if comparable results are subsequently found for 5, 20, or 100 other traits? Would that require that the species be chopped up into a very large number of conservation units, each requiring separate legal protection, recovery plans, etc.? Evolutionary theory suggests that strong associations with a few genes of large effect are more likely when gene flow is relatively high (Yeaman & Whitlock, 2011), which is not uncommon in salmon, and some other large‐effect genes already have been reported in salmon. Pearse, Miller, Abadía‐Cardoso, and Garza (2014) identified a chromosomal inversion that is strongly associated with expression of anadromy in steelhead/rainbow trout. Barson et al. (2015) found a single gene strongly associated with age at maturity in Atlantic salmon, but this was thought to be an exception because it appears to be important in resolving sexual conflict. Veale and Russello's (2017) range‐wide study of sockeye salmon found that reproductive ecotype (spawning either along lakeshore beaches or in streams) is controlled by a single locus. Pritchard et al. (2018) found evidence for diversifying selection in multiple regions of the genome in Atlantic salmon. A comprehensive synthesis of published and unpublished association studies for phenotypic traits in salmonids would be a valuable asset in evaluating this issue.

If strong associations with one or a few genes prove to be relatively common for phenotypic/life history traits in salmon, it would raise the following question with respect to the example considered here: Is there something particularly important about adult migration timing that indicates it should get special consideration for defining conservation units? If one were inclined to give special consideration to one trait above all others, it should be a trait that is of fundamental importance to the ecology and evolution of the species. It might be argued that adult run timing is such a trait, primarily because it increases reproductive isolation and facilitates further local adaptation and divergence (Quinn, Unwin, & Kinnison, 2000), but also because this trait is widely used in management and conservation planning. But this would represent a novel, perhaps unprecedented, approach to applied conservation. Funk et al. (2012) cautioned against focusing on individual traits in defining ESUs; instead, they recommended that both neutral and adaptive genes be used, and that adaptive significance be assessed using a larger suite of markers (“outlier loci”) that show evidence for effects of natural selection across the genome. In this context, Micheletti, Matala, Matala, and Narum (2018) examined associations between >24,000 SNPs and landscape features along migration routes for Columbia River steelhead. GREB1 was one of several dozen SNPs that were both statistical outliers based on *F*_ST_ AND statistically associated with landscape features along migration corridors.

In the end, any decisions about potential changes to management/conservation of Pacific salmon and steelhead will have to consider Question 8 (efficacy of existing management procedures) in light of whatever information becomes available through consideration of the other questions. The current U.S. regulatory framework for salmon involves a combination of federal, state, tribal, and local governance. The states and Native American tribes have primary responsibility for routine management of Pacific salmon and steelhead; this typically focuses on individual populations or stocks. Under the ESA, the federal government takes a broader perspective that focuses on ensuring that major components of ecological/evolutionary diversity (salmon and steelhead ESUs) are conserved.

Most of these ESUs include a dozen or more populations considered to be demographically independent, but more similar to each other than to populations in other ESUs. Assessing overall extinction risk of complex conservation units like these is challenging; for Pacific salmon and steelhead, ESA risk assessments are guided by the four Viable Salmonid Population criteria: abundance, trends, spatial structure, and diversity (McElhany, Ruckelshaus, Ford, Wainwright, & Bjorkstedt, 2000). Evaluating abundance and trends are core features of any risk assessment, and these are conducted separately for each population. Spatial structure and diversity are primarily evaluated at the ESU level in determining how many populations, with what combinations of characteristics, are required for the ESU as a whole to be viable. Formal ESA recovery‐planning teams have partitioned most listed ESUs into multiple strata that reflect various combinations of geography and genetic, ecological and life history diversity, and most plans require viable populations in each stratum before an ESU can be delisted (Waples, McClure, Wainwright, McElhany, & Lawson, 2010). Diversity in adult migration timing typically receives careful consideration in this process (see Hard et al., 2015 and McElhany et al., 2006).

However, detailed analysis of population structure and within ESU diversity has only been conducted during ESA recovery planning for listed ESUs. When the status of an unlisted ESU is evaluated, a key question becomes, “How do various components of spatial structure and diversity contribute to viability of the ESU as a whole?” With respect to adult migration timing, a related question is, “If an early‐migrating population is lost, under what circumstances, and over what time period, might it be restored?” Under the parallel evolution paradigm, the main requirement would be a robust, late‐migrating population to act as an evolutionary source. The time required for evolution to produce a viable population with novel migration timing is not known precisely, but several lines of evidence suggest it might be approximately a century (roughly 25 salmon generations) or less—but only if the environmental conditions that select for different life history traits are present (Fraser, Weir, Bernatchez, Hansen, & Taylor, 2011; Gustafson, Waples, Kalinowski, & Winans, 2001; Hendry, Wenburg, Bentzen, Volk, & Quinn, 2000; Quinn et al., 2000; Waples et al., 2004).

How might this picture change in light of new genomics data? If one takes the large‐effect‐gene hypothesis to its extreme, such that expression of a key phenotypic trait requires a specific gene that is only found in populations that express that phenotype, then evolution of a new population with that phenotype would require immigration of the key genes from another population that already expresses the trait. This could be a real conservation concern, especially if populations that express the trait are rare, declining, or far away. Under this scenario, it might not be sufficient to focus risk analyses independently on one conservation unit at a time. Instead, it could be important to supplement this with a broader perspective to ensure that a sufficient number of potential source populations for the key alleles are maintained across large geographic areas.

The associations between specific alleles and migration timing phenotypes that have been reported to date in Chinook salmon and steelhead are not this extreme. However, the strength of these associations can vary with the method for SNP detection. The studies by Hess et al. (2016) and Prince et al. (2017) used reduced‐representation (RAD) methods that sample only a portion of the genome, leaving open the possibility that the actual associations could be stronger. Thompson et al. (2018) showed that a higher resolution analysis of the GREB1L region can produce stronger phenotype–genotype associations in Chinook salmon. This suggests that our understanding of the relative frequency and importance of large‐effect genes in salmon is likely to continue to evolve in the near future as sampling across the genome becomes more comprehensive.

Compiling more information regarding Question 1 is essential to determine whether the paradigm for thinking about evolution of migration timing diversity needs a major adjustment. If alleles associated with early migration timing are widely distributed in populations that do not have the early migration phenotype, then the parallel evolution paradigm might still be largely applicable in practice, even though the explanatory mechanism would require updating. That is, parallel evolution of early migrating populations from nearby late‐migrating populations based on standing genetic variation might still be the norm, but each realization of this scenario would involve a mix of the same few alleles of large effect and potentially different combinations of many more alleles of smaller effect, such as those found by Brieuc, Ono, Drinan, and Naish (2015).

Conversely, if alleles associated with early migration timing are only found in early migrating populations, then it would be important to ensure that populations with that phenotype persist. What would that mean for conservation units under the ESA (see Figure 2, right panel)? Should each river be a separate DPS that includes both life history types (Figure 2a)? Should each existing DPS be split into two (one with all early migrating populations, the other with all late‐migrating populations; Figure 2b), even though that would be at odds with overall genetic affinities at >99% of the markers surveyed? Should each early migrating population be a separate DPS, with the late migrating populations either lumped or in separate DPSs (Figure 2c,d)? Pros and cons exist for each of these strategies, as well as others that can be imagined. In the end, the effectiveness of conservation and management efforts depends not only on how CUs are defined, but rather on the interaction between the CU structure and the framework for assessing extinction risk. That is, any of the CU frameworks in Figure 2 might potentially provide a template for effective conservation of life history diversity, provided it were coupled with mechanisms to ensure continued opportunities to express key life history phenotypes.

Does the DU framework under SARA allow more flexibility for dealing with this issue in Canada? Perhaps, but whether that actually is the case remains to be seen. The Department of Fisheries and Oceans, Canada (DFO) has defined CUs of Pacific salmon and Atlantic salmon, some of which can be fine‐scaled and include only one adult migration type (e.g., DFO 2013). However, different frameworks are used to define CUs of Pacific salmon (Holtby & Ciruna, 2007) and Atlantic salmon (DFO and MRNF 2008). Furthermore, CUs are not recognized by SARA and receive no federal protection, and there is no formal process to convert CUs into potentially listable DUs. Therefore, how this issue might play out for salmon in Canada is speculative at present. In the example considered in this Perspective, new genomics information has confronted existing conservation and management paradigms regarding how to define conservation units. Somewhat parallel issues would arise in considering how information about large‐effect genes might influence other aspects of applied conservation and management, such as reintroductions, translocations, assisted gene flow, captive propagation, gene banking, and genetic rescue. Most or all of the key questions identified above would be useful in evaluating the relevance of large‐effect genes for these other applications. Furthermore, although we have focused on a specific example involving Pacific salmonids, the issue of how best to use genomics data in conservation is of much broader relevance, as evidenced by lively discussions in the scientific literature (Funk et al., 2012; McMahon, Teeling, & Höglund, 2014; Pearse, 2016; Prado‐Martinez et al., 2013; Primmer, 2009). As demonstrated by Bay et al. (2018), phenological traits roughly comparable to adult migration timing are of considerable ecological and evolutionary importance to a wide range of taxa.

Applied conservation is hard because biology is complex and messy, and every practical application involves interactions with human customs, laws, and institutions that can differ greatly from place to place. This means that each new application typically introduces new wrinkles that have to be considered as special cases. But there is something that is more general and more broadly applicable to a wide range of issues: The *process* involved in figuring out how best to apply genomic data (or any new kind of data) to a real‐world conservation problem. This process involves several steps: (a) providing background and context; (b) identifying what is novel about the new data and what new questions it raises; (c) identifying additional sources of information that can help answer some or all of these questions; (d) discussing how the new data could be used to help address the ultimate question, which is “What is the best way to use the new information in applied conservation?” Outlining this type of process is what we have tried to accomplish in this perspective.

Over time, as the number of real‐world applications of genomics continues to grow, so too will our understanding of the role genomics information can play in 21st Century conservation and management. We are still in the early stages of this with respect to application of genomics data. In the meantime, this overall effort can be enhanced by a systematic approach to work through each applied problem to provide managers and decision makers with as much relevant information as possible upon which to base their decisions.

## CONFLICT OF INTEREST

None declared.
